# First Report of Porcine Parvovirus 2 (PPV2) in Pigs from Colombia Associated with Porcine Reproductive Failure (PRF) and Porcine Respiratory Disease Complex (PRDC)

**DOI:** 10.1155/2024/1471536

**Published:** 2024-05-16

**Authors:** Diana S. Vargas-Bermudez, Marta Mainenti, María F. Naranjo-Ortiz, José Darío Mogollon, Pablo Piñeyro, Jairo Jaime

**Affiliations:** ^1^Facultad de Medicina Veterinaria y de Zootecnia, Departamento de Salud Animal, Centro de Investigación en Infectología e Inmunología Veterinaria (CI3V), Universidad Nacional de Colombia, Sede Bogotá, Carrera 30 No. 45–03, Bogotá, CP 111321, Colombia; ^2^Department of Veterinary Diagnostic and Production Animal Medicine, College of Veterinary Medicine, Iowa State University, Ames, IA, USA

## Abstract

Pigs are affected by various parvoviruses (PPVs); eight have been reported to date (PPV1–PPV8). Porcine parvovirus 1 is considered a primary agent of porcine reproductive failure (PRF), while it is unknown whether other PPVs impact porcine health. Recently, the presence of PPV2 has been confirmed in the lung, either as a single agent or in the form of coinfection with other respiratory; therefore, it has been proposed as a potential participant in the porcine respiratory disease complex (PRDC). In the present study, the presence of PPV2 alone and coinfection with other viruses (PCV2, PCV3, and PRRSV) was evaluated in lung samples obtained from pigs with respiratory signs (respiratory group: RG) (*n* = 146) and stillborn lungs (stillborn group: SG) (*n* = 19) from 82 farms in the five regions with the highest swine production in Colombia. The overall PPV2 prevalence was 37.6% (62/165), with the highest proportion mainly detected in grow-finisher pigs (62.5%), while its herd prevalence was 51.2% (42/82). The most prevalent virus was PRRSV in both groups, while PPV2 alone was found only in the RG group. The most common dual coinfection in the RG and SG was PCV2/PRRSV (17.8% and 10.5%), while the most frequent coinfections involving PPV2 in the RG were PPV2/PCV2 (7.5%) and PPV2/PRRSV (4%) and PPV2/PCV2 (5.3%) in the SG. The most common triple coinfection was PPV2/PCV2/PRRSV at 15% in the RG and 21% in the SG, while quadruple coinfection PVV2/PCV2/PCV3/PRRSV was detected only in the RG (5.5%). Histopathological evaluation of 21 PPV2-positive lungs showed variable degrees of histiocytic or lymphohistiocytic interstitial pneumonia (9%) in the RG, while no significant changes were observed in SG; in addition, neutrophilic bronchopneumonia was observed in 73.7% if cases evaluated. *In situ* hybridization-RNAScope® confirmed the presence of PPV2 within pulmonary lesions in 2/19 RG pigs, while no *in situ* detection was observed in the SG pigs. The phylogenetic evaluation of seven PPV2 sequences detected in Colombia was compared with another 102 reported sequences, indicating that the Colombian strains are located in clade 2. Our results confirm the presence of PPV2 in pigs with PRDC alone and pigs coinfected with PCV2, PCV3, and PRRSV. Likewise, its presence alone or in coinfection in stillbirths suggests that PPV2 is also involved in PRF.

## 1. Introduction

Parvoviruses are small naked viruses belonging to the family *Parvoviridae* and containing an ssDNA genome of approximately 4–6 kb [1]. Pigs can be infected by various genera of porcine parvoviruses (PPVs); specifically, porcine parvovirus 2 (PPV2) belongs to the genus *Tetraparvovirus* [2]. This parvovirus was first discovered incidentally in serum samples of pigs tested for hepatitis E virus [3], and since then, it has been reported in Asia [4–6], Europe [7–10], South Africa [11], North America [12–14], and South America, particularly Brazil [15]. Despite recent reports of PPV2 detection, phylogenetic and phylodynamic studies in Europe have determined that PPV2 must have been circulating since at least the 1920s [8]. Based on these reports, it can be inferred that PPV2 is present worldwide and that it has been detected in multiple types of swine samples at various prevalence rates: feces 6%–18% [16], serum 5.4%–54% [7, 16], tonsils 28%–100% [5, 9, 10], hearts 55% [9], lungs 30%–42.7% [10, 17], nasal swabs 0.3%–2% [10, 18], oral fluids 48% [16], spleen 43% [10], lymph nodes 30%–52% [10], and fetal tissues 2.8%–98% [10, 14]. Prevalence rates also varied among pigs at different production phases: piglets 2%–67% [10, 18, 19], prefattening 11%–30% [10, 19], and fattening 20%–68% [10, 16, 19]. Current information regarding PPV2 pathogenesis, cellular tropism, clinical manifestations, and diagnosis is scarce. Recent reports have proposed that PPV2 may be a participant in the porcine respiratory disease complex (PRDC) [18, 20–22] and cause porcine reproductive failure (PRF) [14]. Notably, in a study conducted in Hungary, high levels of anti-PPV2 antibodies were detected in 40–50-day-old pigs with PRDC on a farm negative for porcine reproductive and respiratory syndrome virus (PRRSV) and had low levels of antibodies for both porcine circovirus type 2 (PCV2) and swine influenza virus (SIV) [23]. In other studies from the US and China, higher detection rates or higher quantities of PPV2 DNA were detected in serum or lung samples from prefattening and fattening pigs with respiratory disease [12, 13]. On the other hand, PPV2 is often detected in conjunction with other respiratory pathogens, particularly PCV2 and PRRSV, suggesting that it may be acting in coinfection in cases of porcine circovirus-associated disease (PCVAD) and PRDC [10, 20]. Although the pathogenesis of PPV2 infection has not been thoroughly evaluated, histological evaluation of lungs from PCR-positive animals has linked PPV2 detection with interstitial pneumonia. In addition, direct detection in tissues by *in situ* hybridization (ISH) has demonstrated PPV2 replication mainly in alveolar macrophages and lymphocytes, suggesting cellular tropism for the monocyte macrophage and lymphocytic lineages [21, 22].

Therefore, the objectives of this study are to (1) to determine the presence of PPV2 in respiratory cases and cases of reproductive failure, (2) to associate the presence of PPV2 with potential coinfection with other viral pathogens, (3) to evaluate the severity of the pulmonary lesions associated with PPV2 infection, (4) to evaluate the presence of PPV2 within pulmonary lesions by *in situ* hybridization, and (5) to evaluate the genetic diversity of strains potentially circulating in Colombia.

## 2. Materials and Methods

### 2.1. Sample Collection

**Figure 1:**
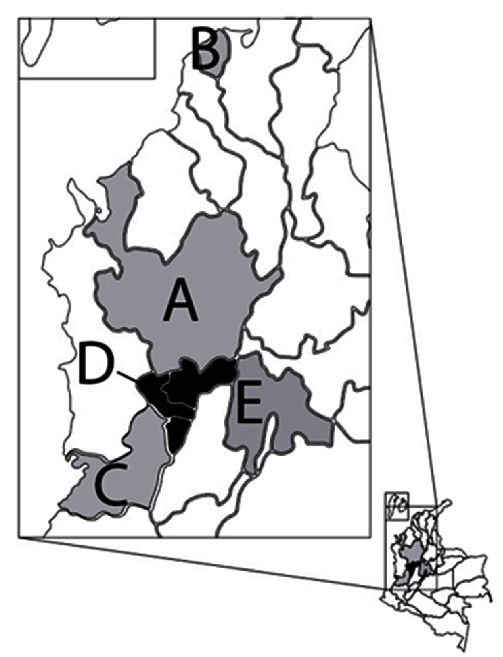
Map of Colombia indicating the five provinces sampled in the study and their geographical relation with the areas of high swine density in the country. (A) Antioquia, (B) Atlántico, (C) Valle del Cauca, (D) Eje Cafetero, and (E) Cundinamarca. In black is highlighted the Eje Cafetero region that includes three provinces (Caldas, Risaralda, and Quindío). The map was downloaded from the National Department of Statistics of Colombia (DANE) database (http://geoportal.dane.gov.co/acerca-geoportal/acerca/), and the results were adapted to the map using the QGIS software available online (https://qgis.org/es/site/).

Samples were collected between 2021 and 2022 from 82 swine farms strategically located across the five primary regions with the highest swine production in Colombia (Antioquia, Valle del Cauca, Cundinamarca, Atlántico, and Eje Cafetero; Figure 1(A)–1(E)). The criteria for sample selection were based on the presence of respiratory clinical signs (dyspnea, tachypnea, and cyanosis) defined as the respiratory group (RG) and cases of reproductive failure in sows or gilts with the presence of the stillborn defined as the stillborn group (SG). A total of 165 lung tissue samples were collected, with 146 in the RG originated from nursery or grow-finisher pigs exhibiting respiratory signs and the remaining 19 in the SG obtained from stillborn pigs. The RG subset included 3–8-week-old nursery pigs (*n* = 122) and 8–23-week-old grow-finisher pigs (*n* = 24).

### 2.2. Sample Processing and Viral Extraction

Lung tissues were minced and diluted at 1 : 10 (weight/volume) in phosphate-buffered saline (PBS) (pH ∼ 7.4), homogenized, and centrifuged at 1,500x *g* for 10 min. Total nucleic acid was extracted from the supernatant using a pure viral nucleic acid kit (Roche®) according to the manufacturer's instructions. All extracts were stored at −80°C until RT-PCR evaluation.

### 2.3. Detection of PPV2 and Other Viruses Associated with PRDC and PFP via Real-Time PCR

The PPV2-NS1 region was amplified via real-time PCR using primers Q1 (5′-GCGCATTCGCCAAACTAGCTC-3′) and Q2 (5′-GTTTGCCCTTAATGTCGATCC-3′), as previously reported by [5], obtaining a product of 187 bp. Amplicons obtained from PPV2-positive samples were cloned into TOPO-TA (ThermoFisher Scientific®) according to the manufacturer's instructions. The PPV2-NS1 recombinant plasmid was sequenced by a commercial sequencing company, SSiGMol (Servicio de Secuenciación y Análisis Molecular, Instituto de Genética, Universidad Nacional de Colombia, Bogotá). The PCR products were directly sequenced in both directions (SSiGMol). The reactions were performed in a volume of 20 *μ*L containing 50 ng of DNA, 10 *μ*L of 2X SsoAdvanced Universal SYBR Green Supermix (Bio-RAD®), and 0.4 *μ*M of each primer. The assay was performed using a Light Cycler® 480 II-Roche thermal cycling system under the following cycling conditions: 95°C for 5 min, followed by 40 cycles of 95°C for 1 min, 60°C for 25 s, and 72°C for 5 s. Melting curves were generated by monitoring the SYBR green signal's fluorescence from 70°C to 95°C. The negative control contained ddH_2_O, and the positive control was the PPV2-NS1 plasmid. All reactions were conducted in triplicate. The 10-fold serial dilutions were evaluated as the limit of detecting the SYBR green-based real-time qPCR from 1 × 10^2^ to 1 × 10^9^ copies/*μ*L. The quantification cycle (Cq) values range from 7.5 to 35 cycles, with a linear correlation (R2) of 0.979 (slope = −3,329) between the Cq value and the logarithm of the PPV2 copy numbers. Therefore, samples with no cycle threshold (Ct) at 36 cycles were considered negative. The specificity of qPCR for PPV2 was confirmed by using DNA samples that were positive for other DNA viruses, including PCV2, PCV3, and PPV1. Additionally, all samples were tested for PCV2, PCV3, and PRRSV via qPCR. The sequences of the primers used to detect these viruses are described in Table S1.

### 2.4. PPV2 Sequencing and Phylogenetic Analysis

A combination of five overlapping primers was used to perform a genetic characterization of PPV2 strains circulating in Colombia. Primers previously described [5] and newly designed were based on sequences from different countries published on GenBank. Amplification primers for this study were generated using the Primer 3 input (v3.0.0; Institute for Biomedical Research, Boston, MA) [24]. All primer sequences and PPV2 genome target regions are listed in Table 1. Amplification reactions were performed in a total volume of 25 *μ*L containing 0.25 *μ*L of Accu Prime Taq (5 U/mL) (Thermo Fisher®), 1x AccuPrimer PCR buffer (2.5 *μ*L), 1 *μ*L of each primer (20 *μ*mol), and 2 *μ*L of extracted DNA. Briefly, PCR conditions included denaturation at 94°C for 2 min, followed by 35 cycles of denaturation at 94°C for 30 s, annealing at 57°C for 30 s, and extension at 68°C for 1 min. All reactions were performed on a BioRad®-DNA thermocycler. The PCR products were directly sequenced in both directions (SSiGMol). Nucleotide BLAST (basic local alignment search tool) was used to search the sequences available in the NCBI nucleotide database. Seven partial genome sequences (nt 380–4333) were obtained, which included the PPV2-NS and VP genes. Genomic alignment was carried out with the ClustalW method and compared with other PPV2 nucleotide sequences retrieved from GenBank. The best-fit model for nucleotide substitution was identified as a Tamura 3 parameter model with gamma-distributed heterogeneity and invariable sites (T92 + G + I). A phylogenetic reconstruction was performed using the maximum-likelihood (ML) method, with a bootstrap value of 1,000. The best model and phylogenetic analysis were performed by using MEGA™ 7.0 for Windows® [25]. The robustness of the ML trees was statistically evaluated by bootstrap analysis with 1,000 bootstrap samples.

### 2.5. Histopathology, Immunohistochemistry (IHC), and *In Situ* Hybridization (ISH)

The characterization of pulmonary lesions and PPV2 direct detection in tissues were performed in a subset of 21 pigs (19 RG and 2 SG) PPV2 positive by PCR where fixed tissues where available. Lung tissue samples were fixed in 10% neutral buffered formalin, embedded in paraffin (FFPE), and routinely processed for histologic examination. Approximately 3−5-micron-thick sections were stained with hematoxylin and eosin (HE) and reviewed by two diagnostic pathologists at the Iowa State University Veterinary Diagnostic Laboratory in Ames, Iowa, using an Olympus BX43 bright-field microscope (Olympus Corporation, Tokyo, Japan). Histologic lung lesions were scored according to their distribution within the tissue section (1 = focal; 2 = multifocal; 3 = diffuse) and the severity of each lesion (0 = absent; 1 = mild; 2 = moderate; 3 = severe).

For the PPV2 *in situ* hybridization (ISH) and PRRSV and PCV2 immunohistochemical analyses, one lung tissue core with a diameter of 5 mm was extracted from each selected FFPE block and transferred to a recipient paraffin block containing a total of six tissue cores. Approximately 3−5-micron-thick sections were obtained from each recipient block. The *in situ* hybridization was developed through the RNAScope® platform (Advanced Cell Diagnostics, Newark, CA, USA), targeting the specific reverse complementary nucleotide sequence of the capsid gene (nt 3684–4579). Positive hybridization signals represent active viral transcription characterized by detection of the PPV2 mRNA-encoding capsid protein. The RNAScope® positive control probe of *Sus scrofa* peptidylprolyl isomerase B (Sc-PPIB; catalogue no. 428591), which targets the eukaryotic PPIB gene, and a negative control probe of *Bacillus subtilis* dihydrodipicolinate reductase (*DapB*) gene (catalogue no. 310043) were designed and synthesized by Advanced Cell Diagnostics. After hybridization, the slides were counterstained with a 50% hematoxylin solution. The PRRSV and PCV2 immunohistochemistry was performed as previously described [26, 27].

## 3. Results

### 3.1. Prevalence of PPV2 and Other Viruses Associated with PRDC and PFP

**Figure 2:**
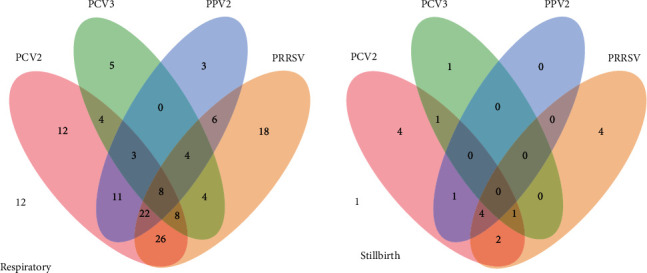
Venn diagram representing the distribution of the viruses detected (PPV2, PCV2, PCV3, and PRRSV) by PCR and RT-PCR in on lung samples in pigs with respiratory symptoms (RG group) and stillborn (SG group).

The PPV2 detection by qPCR in 165 lungs combining nursery and grow-finisher pigs (RG) and stillborn pigs (SG) showed an overall detection rate of 37.6% (95% CI; 30.1%–44.9%) (62/165) and a 51.2% positive farm rate (95% CI; 39.9%–62.4%) (42/82). Regardless of coinfection with other viruses, the PPV2 detection rate was 39.0% (95% CI; 31.1%–47.4%) (57/146) and 26.3% (95% CI; 9.1%–51.2%) (5/19) in the RG and SG, respectively. More specifically, PPV2 was detected in 34.4% (42/122) of nursery pigs and 62.5% (15/24) of grow-finisher pigs (Tables 2 and 3). Geographically, PPV2 was detected in four (Antioquia, Cundinamarca, Eje Cafetero, and Valle del Cauca) of the five regions evaluated in this study. In addition to the detection of PPV2 in the lungs of RG and SG piglets, multiple other pathogens were detected, including PCV2 in 64.8% (95% CI; 57.0%–72.1%) (107/165), PRRSV in 64.8% (95% CI; 57.0%–72.1%) (107/165), and PCV3 in 23.6% of piglets (95% CI; 17.4%–30.1%) (39/165). The overall detection rates for PCV2, PCV3, and PRRSV, as well as the proportion of negative animals in each group age, are presented in Table 2. Regardless of the pathogen detected, cases in which only one pathogen was detected were classified as a single infection. Overall, 26% (38/146) and 47.4% (9/19) of the lungs evaluated in the RG and SG, respectively, showed a single pathogen infection. Interestingly, among cases with a single pathogen detection, PPV2 was confirmed in 2.1% (3/146) of the lungs in the RG, and no single PPV2 cases were detected in the SG. The proportions of cases presenting single PCV2, PCV3, or PRRSV infection are summarized in Table 3. Cases in which two simultaneous pathogens were detected were classified as coinfection (dual). Specifically, 34.9% (51/146) and 21.0% (4/19) of the lungs evaluated in the RG and SG, respectively, showed dual pathogen infection. The proportion of PPV2 dual coinfection in the RG includes PPV2/PCV2 at 7.5% (11/146), PPV2/PRRSV at 4.1% (6/146), and no PPV2/PCV3. The coinfection rate detected in the SG only showed 5.3% (1/19) of PPV2/PCV2 cases, and no other dual coinfections were observed. The proportion of cases presenting dual coinfection without PPV2 is summarized in Table 3. Cases in which three pathogens were detected simultaneously were classified as coinfections (triple). Specifically, 25.3% (37/146) and 26.3% (5/19) of the lungs evaluated in the RG and SG, respectively, showed triple pathogen infection. The most common triple coinfections in which PPV2 was involved in the RG were PPV2/PCV2/PRRSV, at 15.1% (22/146); PPV2/PCV3/PRRSV, at 2.7% (4/146); and PPV2/PCV2/PCV3, at 2.1% (3/146). Only PPV2/PCV2/PRRSV coinfection was observed in the SG, at 5.3% (1/19). Finally, quadruple coinfection with PPV2/PCV2/PCV3/PRRSV was observed in the RG in 5.5% (8/146) of samples evaluated (Figure 2 and Table 3). Based on the Ct values, the amount of virus detected was variable, with an average Ct value of 27.1 for PPV2 (min 15; max 30), 30.6 for PCV2 (min 16; max 35), 30.7 for PRRSV (min 25.5; max 34.2), and 31.2 for PCV3 (min 24; max 34).

### 3.2. Histopathology, ISH, and IHC

**Figure 3:**
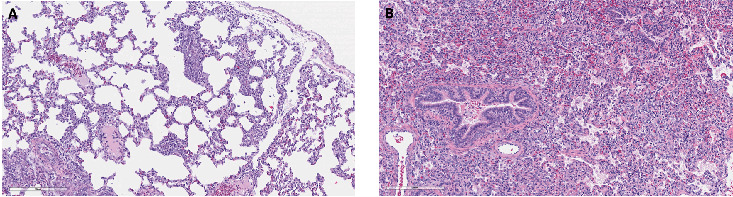
Histological changes associated with PPV2 infection. A representative section of lung of a single PPV2-positive RG case with variable degrees of histiocytic and lymphohistiocytic interstitial pneumonia with alveolar thickening due to infiltration of lymphocytes and macrophages (A). Other histological changes observed in the PPV2-positive lungs from the RG include neutrophilic bronchopneumonia (B).

**Figure 4:**
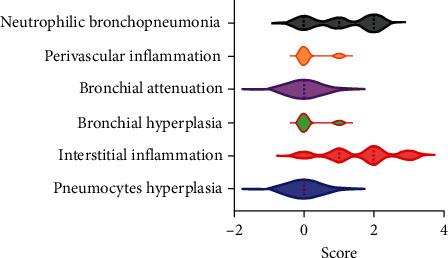
Distribution of histological lesions score on a subset of RG pigs (*n* = 19). Histologic lesions in lungs were scored according to the distribution within the tissue section (1 = focal; 2 = multifocal; 3 = diffuse) and degree of each lesion recorded (0 = absent; 1 = mild; 2 = moderate; 3 = severe).

Distribution of histological lesions was scored on a subset of the RG PPV2 positive representing single, dual, and triple coinfections where fixed tissues were available. The histologic evaluation of 21 pigs showed variable degrees of histiocytic and lymphohistiocytic interstitial pneumonia in 94.7% (95% CI; 73.9%–99.8%) (18/19) of pigs from the RG (Figure 3(A)), while no significant changes were observed in the two stillbirths of the SG. Other histological changes observed in the PPV2-positive lungs from the RG include neutrophilic bronchopneumonia in 73.7% (95% CI; 48.8%–90.8%) (14/19) of cases, indicating a high proportion of bacterial coinfection (Figure 3(B)). Other histological changes included mild hyperplasia of the bronchial epithelium in 15.8% (95% CI; 3.4%–39.6%) (3/19) and perivascular inflammation in 26.3% (95% CI; 6.5%–51.2%) (5/19) of the PPV2-positive cases in the RG. The distribution of the lesions was mainly diffuse in 42.2% of cases (95% CI; 20.0%–66.5%) (8/19) or multifocal in 47.3% of cases (95% CI; 24.9%–69.8%) (9/19), and rarely focal in 5.3% of cases (95% CI; 0.1%–26.0%) (1/19). The distribution of scores based on the severity and type of lesions in this subset of RG pigs is shown in Figure 4.

**Figure 5:**
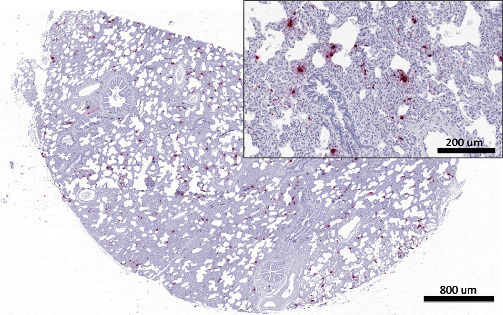
Lung, pig. *In situ* detection of PPV2-mRNA by *in situ* hybridization (RNAScope® platform). Viral mRNA signal was detected multifocally in the alveolar septa of a nursery pig with respiratory signs. Positive signal is labeled in red. Hematoxylin counterstain.

The presence of PPV2 within pulmonary lesions was evaluated via ISH-RNAScope® in 21 cases that were confirmed PPV2 positive based on PCR. A positive PPV2 mRNA signal was confirmed within the alveolar septa of 2/19 RG animals (Figure 5), and there was no *in situ* detection on positive PCR samples in the SG. In addition, in the same group of tissues, PCV2 was detected in 84.2% (95% CI; 60.4%–96.6%) (16/19) and PRRSV in 68.4% (95% CI; 43.4%–87.4%) (13/19) of cases among the RG animals and both animals from the SG. However, neither PCV2 nor PRRSV was confirmed by immunohistochemistry in any of the evaluated lungs in the RG or SG.

### 3.3. Sequence Analysis of PPV2

Of the 65 PPV2-positive lung samples, a nearly complete genome (5,215 nucleotides) was obtained in seven cases. The nucleotide region confirmed by the sequence comprises 5,215 nucleotides (nt) with incomplete reads of 233 nt of the ITR-5`region and 84 nt of ITR-3`. Information, including geographic origin, sample collection date, access number, and the clinical syndrome of the affected animals for the seven confirmed PPV2 sequences, is presented in Table S2. Multiple alignments of the seven Colombian sequences showed an nt identity ranging from 98.1% to 99.7%. Comparing these seven sequences with the 102 nearly complete PPV2 genome sequences reported in GenBank showed an nt identity ranging from 93.2% to 99.9%. The ORF1 region of the seven sequences identified in this study had a length of 1986 nt, encoding 662 amino acids (aa) of the nonstructural protein NS. Among these seven strains, ORF1 showed a proportion of identity at the nucleotide level of 97.7%–99.8%, as well as 98.3%–100% at the aa level. The ORF2 region of the sequences indemnified in this study had a length of 3099 nt, encoding 1,033 aa of the VP structural protein. The identity of the ORF2 region varied from 98.4% to 99.7% at the nucleotide level and 94.1%–100% at the aa level.

**Figure 6:**
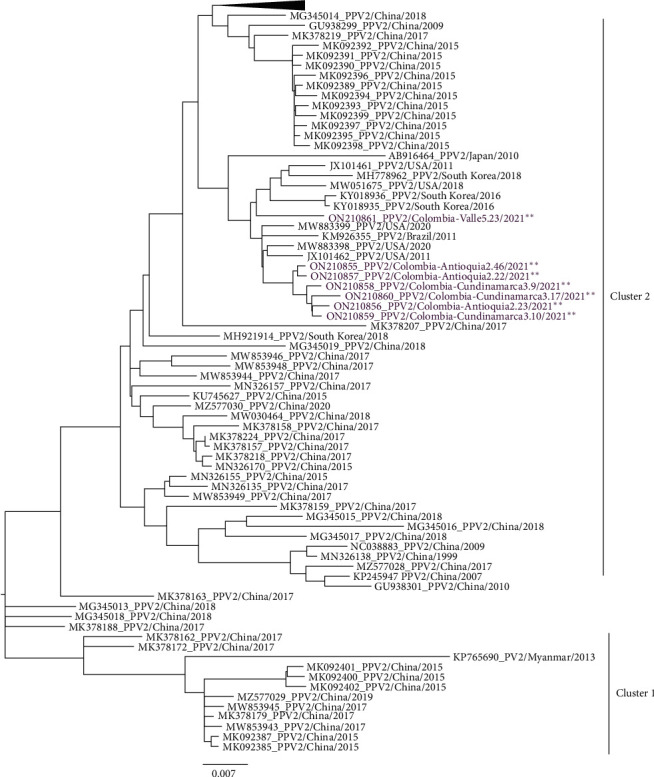
Maximum likelihood phylogenetic tree of PPV2 inferred based on the alignment of near-complete genome sequences (NCG). Sequences in blue and  ^*∗∗*^ indicated the positions of seven Colombian sequences from the present study in the tree. The 102 PPV2-NCG (5125 nt) reported in the GenBank database were included in the phylogenetic analysis. The phylogenetic tree was constructed by ML analysis using the Tamura 3 parameter with gamma distribution with tree topology evaluated with 1,000 bootstrap replicates.

The phylogenic analysis of 109 sequences of nearly complete genomes, including both ORF1 (NS) and ORF2 (VP) regions (seven obtained in this study and 102 global sequences), is distributed into two main clades. Clade 1 contains sequences from Asia (China and Myanmar), and clade 2 contains sequences from China, South Korea, Japan, the United States, and Brazil. Additionally, clade 2 is subdivided into seven to eight subclades depending on the region of the genome being analyzed (nearly complete genome, ORF1 or ORF2). The Colombian sequences are aligned in the same subclade of sequences reported in Japan, South Korea, the US, and Brazil (Figure 6). The Colombian PPV2 strains were designated PPV2/Col/Valle5.23/2021, PPV2/Col/Antioquia2.46/2021, PPV2/Col/Antioquia2.23/2021, PPV2/Col/Antioquia2.22/2021, PPV2/Col/Cundinamarca3.9/2020, PPV2/Col/Cundinamarca3.10/2021, and PPV2/Col/Cundinamarca3.17/2021 and stored in GenBank under accession numbers ON210855 to ON210861.

## 4. Discussion

Since its first detection in 2001, there has been a growing interest in understanding the participation of PPV2 in different clinical syndromes in pigs. Based on recent reports, PPV2 has been suggested to be involved in PRDC and PRF syndromes, which are associated with high economic losses in the swine industry [28–30]. PRCD is defined as a polymicrobial respiratory disease caused by various viruses and bacteria, typically with primary pathogens inducing lesions in the respiratory system, which would favor infection with secondary or opportunistic pathogens [31]. Well-established primary viral pathogens in respiratory syndromes in pigs include PCV2, PRRSV, and SIV, but some novel candidates have recently been proposed, such as PCV3 [32, 33]; porcine coronavirus [34, 35]; and novel PPVs, including PPV2 [20–22, 31]. For PRF, primary pathogens include PPV1, PCV2, and PRRSV [36], and recent candidates are PCV3 [37] and some novel PPVs [14]. The presence of primary pathogens varies between and within farms in different age groups, as well as between regions and countries; therefore, it is not possible to establish a standard etiologic profile, making it necessary to determine the participating pathogens in each case [31].

This is the first study to demonstrate the presence of PPV2 in Colombia. Specifically, PPV2 was found to be widely distributed in four (Antioquia, Cundinamarca, Eje Cafetero, and Valle del Cauca) of the five regions with the highest swine production in the country. Prior to this study, PPV2 was only reported within South America, in Brazil [15], as well as in Latin America, in Mexico [14].

In our study, we evaluated the presence of PPV2, along with three additional viral agents (PCV2, PCV3, and PRRSV), in lung tissue from pigs with clinical respiratory signs (RG; *n* = 146) or stillbirth (SG; *n* = 19) at 82 swine farms in Colombia. Regardless of the clinical syndrome, the most prevalent viruses were PRRSV and PCV2, which is consistent with previous studies indicating these agents to be the primary agents in PRDC and PRF, either individually [38] or in viral coinfection [18]. An important finding was that PPV2 was the third most prevalent agent in both the respiratory (39%) and reproductive groups (26%). Similar PPV2 prevalence rates in lung samples from diagnostic cases were previously reported in the US (39%) and Korea (32%) [22, 39], while even higher prevalence rates were found in other studies in North America (75.3%) and China (73%), especially in pigs with PCVD (16,17). Moreover, similar to previous reports, PPV2 was found in a higher prevalence in grow-finisher pigs (62%) [10, 12, 13, 22].

Within the respiratory group, the most common single infections were PRRSV and PCV2 (66% and 64%, respectively), while single infections with PCV3 or PPV2 were uncommon (3% and 2%, respectively). It is important to note that the prevalence of single infections in the RG was lower (26%) compared to multiple infections (66%), confirming the notion that the PRDC is the most commonly a multietiological entity, with presentations of coinfections or superinfections [40]. Specifically, dual coinfections were the most prevalent (34.9%), and while PRRSV/PCV2 was the most common combination overall (17.8% of total RG cases), PPV2 triple infection with PCV2 and PRRSV (15.1%) and dual infection with PCV2 (7.5%) were the second and third most common combinations, respectively. The detection of PPV2 was more frequent in PCV2-infected pigs (77.2% of PPV2-positive cases) and PRRSV-infected pigs (70.1%) than in coinfection with PCV3 (26.3%) or as a single infection (5.2%). These findings are consistent with previous reports that detected PPV2 mostly in association with PCV2 [10, 17, 18, 41] or PRRSV infection [42]. Interestingly, PPV2 was found in multiple combinations of coinfection in our study, including triple infections (PPV2/PCV2/PRRSV; PPV2/PCV3/PRRSV; PPV2/PCV2/PCV3; cumulative 19.86%) and quadruple infections (5.48%). Reports on triple and quadruple coinfections involving PPV2 are scarce in the literature. A retrospective study of PCV2 coinfections with novel PPVs from Great Britain [10] found PCV2/PPV2/PPV3 coinfection to be the most prevalent (17%), while other triple coinfections of PPVs and PCV2 were below 1% incidence; however, this study did not include PRRSV codetection rates.

In the SG (*n* = 19), PCV2 and PRRSV were the agents most frequently detected (68% and 58%, respectively), while PPV2 and PCV3 were detected in 26% and 16% of pigs, respectively. Single infections occurred in the SG more commonly than in the RG (47% versus 25%), and the overall prevalence of PPV2 was lower in the SG compared to the RG. The most common single infections in stillbirths were PCV2 (21%) and PRRSV (21%), while single infection with PPV2 was not detected. Interestingly, PPV2 was most commonly found in triple infections with PCV2 and PRRSV (21.1%), rather than in dual infections (5.26%). There are very few reports of PPV2 detection in porcine fetuses. Interestingly, a study conducted in the US in 2013 [12, 13] detected PPV2 via PCR in fetal thoracic fluid of 1/75 pigs (1.3%) but not in lung tissue, while a recent study from Mexico [14] detected PPV2 via PCR in the hearts of 96% of 100 fetuses, which 56 of them were coinfected with PCV2. In our study, PPV2 was detected in 26% of the fetal lungs and only in coinfection with either PCV2 or PRRSV.

In this study, the prevalence of multiple coinfections was lower in SG than in RG pigs, indicating that more diverse combinations of coinfections, including PPV2, occur in respiratory cases compared to reproductive failure cases. The higher prevalence of PPV2 in pigs with respiratory signs in our study suggests that PPV2 may have a more common aerogenous transmission affecting the respiratory tract and, therefore, more frequently involved in PRDC than RFP. Overall, our findings in the RG suggest that PPV2 participates in the PRDC, most likely enhancing and exacerbating lesions induced by primary pathogens, especially when pigs are infected with PCV2 or PRRSV. While the absence of PPV2 detection in the SG, other than in coinfection with other primary viral agents, does not support the role of PPV2 as a primary cause of porcine reproductive failure, its detection by PCR in fetal lungs suggests potential vertical transmission.

Most of the lung tissues from the RG that were evaluated histologically presented variable degrees of interstitial pneumonia (18/19), while no lesions were detected in lung tissues obtained from stillbirths (0/2). Neutrophilic bronchopneumonia consistent with a secondary bacterial infection was also often present (14/19). Although interstitial pneumonia is a lesion commonly attributable to viral infection, the codetection of PCV2 and/or PRRSV along with PPV2 via PCR in 95% of the samples evaluated histologically makes it difficult to attribute these lesions to one specific agent. Interestingly, while PCV2 and PRRSV were not detected via immunohistochemistry in any of the evaluated samples, the presence of PPV2 within the affected alveolar septa was corroborated in 2/10 RG pigs. The lack of immunohistochemical and ISH detection may have been due to the multifocal nature of the infection which can skewer the histological evaluation due to the lack of representation of the main lesions on the section collected in the field, or the low quantity of PCV2 and PRRSV viral antigen because, in most of the evaluated cases, the PCR Ct values for these agents were above 26, suggesting a low viral load.

The most variable portion of the genome is the VP, according to PPV2 sequencing studies; therefore, most of the reports focus on this region [5]. Given this criterion, seven PPV2 clades named A–G [8] were established in Japan. In 2015, Sun et al. [20] analyzed a nearly complete genome, classifying PPV2 into two clusters or clades: cluster 1 includes sequences from Myanmar, and cluster 2 includes sequences from China. In our study, a phylogenetic analysis of a partial PPV2 genome (5,215 nt) was performed, in which including complete ORF1 and ORF2 sequences were compared with sequences in which these entire regions were obtained from Japan, Germany, Mexico, Poland, and Thailand. The phylogenetic analysis of the strains detected in Colombia and 102 PPV2 genomes reported in Genbank demonstrated that the sequences clustered in two different clades. Clade 1 contains the first sequence of PPV2 from Myanmar and sequences from China; clade 2 contains the sequences from China, South Korea, the US, Brazil, and Colombia. It is essential to highlight that 81% of the reported PPV2 sequences were obtained from China. Additionally, we found several subclades within clade 2, including one with sequences of different geographical origins than China. These sequences are from Colombia, the US, Brazil, and South Korea, which suggests that they may have a common origin. Regarding the genetic diversity of the strains, it was observed that all strains detected in Colombia are within a specific subclade within clade 2, where only PPV2 strains from countries other than China are represented.

## 5. Conclusion

This study demonstrates that PPV2 is widely prevalent in pigs in Colombia, most commonly in coinfection with other primary viral agents that are part of the PRDC or PRF, particularly PCV2, and PRRSV. Since cases in this study had a high proportion of coinfection, further investigations are warranted to validate if PPV2 genetic diversity is not associated with different phenotypes. In addition, further studies are necessary to elucidate the pathogenesis of PPV2 infection. Our study warrants that PPV2 diagnosis should be based on a combination of diagnostic techniques, including PCR, ISH, and histologic evaluation, to help understand its role in routine respiratory and reproductive cases.

## Figures and Tables

**Table 1:** Set of primers designed for amplification of PPV2 genome from nt 380 to 4333.

Primer	Sequence	Product length (bp)	GenBank position/accession number
PPV2 S1-Ry	CCGACAGGATAAGTGTCGAG	1,246	151-1397/MW853943^a^
PPV2 S1-Q2	GTTTGCCCTTAATGTCGATCC		

PPV2 S2F	CCTCTCAATCCCTGATTTGG	1,137	1340-2477/MW853943^a^
PPV2 S2R	GTAAAGCTGTAACAGAGCCTGCC		

PPV2 S3F	GGATCAATGCCCTTCTGAGG	973	2361-3334/MW853943^a^
PPV2 S3R	GGTAAGACTTTGTCCAGCTCC		

PPV2 S4F	GGACACGTTAGCGGATCTCAT	1,003	3340-4343/MW853943^a^
PPV2 S4R	GGTTATGACGGAGGTTCAACG		

PPV2-S5-Cnvirus	TTACGAGTTTCCCAGTCTCG	1,150	4273-5423/MW853943^a^
PPV2-S5-Ry	CCGACAGGATAAGTGTCGAG		

^a^GenBank reference sequence. Primers PPV2 S1 and S5 (Ry, Q2, and Cnvirus) were obtained from [5]; primers PPV2 S2, PPV2 S3, and PPV2 S4 were designed.

**Table 2:** Prevalence of PPV2 detection by PCR in pig lungs by age group.

Age group	*N*	Positive	(%)	95% CI
Nursery pigs (3–8 weeks)	122	42	34.4	26.0–43.6
Grow-finisher pigs (8–23 weeks)	24	15	62.5	40.6–81.2
Stillbirths	19	5	26.3	9.1–51.2
Total	165	62	37.6	30.2–45.4

**Table 3:** Detection rate of single and coinfections in nursery and grow-finish pigs (RG) and stillborn pigs (SG).

	Respiratory group (RG)			Stillborn group (SG)		
	*N*	(%)	95% CI	*N*	(%)	95% CI
Total	146	100	97.5–100	19	100	82.3–100
PPV2-positive	57	39.0	31.1–47.5	5	26.3	9.1–51.2
PCV2-positive	94	64.4	56.0–72.1	13	68.4	43.4–87.4
PCV3-positive	36	24.7	17.9–32.5	3	15.8	3.4–39.6
PRRSV-positive	96	65.8	57.4–73.4	11	57.9	33.5–79.7
Negative	12	8.2	4.3–13.9	1	5.3	1.3–26.0
Single infections	38	26.0	19.1–33.9	9	47.4	24.4–71.1
PPV2	3	2.1	0.4–5.9	0	0	0
PCV2	12	8.2	4.3–13.9	4	21.1	6.0–45.6
PCV3	5	3.4	1.1–7.8	1	5.3	0.1–26.0
PRRSV	18	12.3	7.5–18.8	4	21.1	6.0–45.6
Coinfections (dual)	51	34.9	27.2–43.2	4	21.1	6.0–45.6
PPV2/PCV2	11	7.5	3.8–13.1	1	5.3	1.3–26.0
PPV2/PCV3	0	0	0	0	0	0
PPV2/PRRSV	6	4.1	1.5–8.7	0	0	0
PCV2/PCV3	4	2.7	0.8–6.9	1	5.3	1.3–26.0
PCV2/PRRSV	26	17.8	11.9–24.9	2	10.5	1.3–33.1
PCV3/PRRSV	4	2.7	0.8–6.9	0	0	0
Coinfections (triple)	37	25.3	18.5–33.2	5	26.3	9.1–51.2
PPV2/PCV2/PCV3	3	2.1	0.4–5.9	0	0	0
PPV2/PCV2/PRRSV	22	15.1	9.7–21.9	4	21.1	6.0–45.6
PPV2/PCV3/PRRSV	4	2.7	0.8–6.9	0	0	0
PCV2/PCV3/PRRSV	8	5.5	2.4–10.5	1	5.3	0.1–26.0
Coinfections (quadruple)						
PPV2/PCV2/PCV3/PRRSV	8	5.48	2.4–10.5	0	0	0

## Data Availability

The data that support the findings of this study are available from the corresponding author upon reasonable request.

## Supplementary Materials

Supplementary Materials Table S1: sequences of the primers used for the detection of PCV2, PCV3, and PRRSV in this study. Table S2: information about PPV2 sequences obtained from PPV2-positive lung tissue samples from three regions with high swine production in Colombia.
