# Supplementary material for: First Report of Porcine Parvovirus 2 (PPV2) in Pigs from Colombia Associated with Porcine Reproductive Failure (PRF) and Porcine Respiratory Disease Complex (PRDC)
Source: Transbound Emerg Dis. 2024 May 16;2024:1471536. doi: 10.1155/2024/1471536 (PMC12017240; doi:10.1155/2024/1471536)
Supplement: Supplementary Materials — Table S1: sequences of the primers used for the detection of PCV2, PCV3, and PRRSV in this study. Table S2: information about PPV2 sequences obtained from PPV2-positive lung tissue samples from three regions with high swine production in Colombia. [file 1471536.f1.docx]

**Table S1**. Sequences of the primers used for the detection of PCV2, PCV3, and PRRSV in this study.

| Primer/Probe | Nucleotide sequence (5´-3´) | Amplified | Position / Genbank accession number | Reference |
| --- | --- | --- | --- | --- |
| PCV2abF  PCV2abR  ProbePCV2a  ProbePCV2b/d | GCAGGGCCAGAATTCAACC  GGCGGTGGACATGATGAGA  FAM-GGGGACCAACAAAATCTCTATACCCTTT-BHQ  Cy5-CTCAAACCCCCGCTCTGTGCCC-BHQ | 123 | 1411-1534/MZ747121a | [(Tanja Opriessnig et al., 2010)](https://sciwheel.com/work/citation?ids=11565316&pre=&suf=&sa=0&dbf=0) |
| PCV3F  PCV3R  Probe PCV3 | AGACGACCCTTATGCGGAAA  AACGGTGGGGTCATATGTGTTG  FAM-CTCACCCAGGACAAAGCCTCTTCTT-BHQ | 155 | 1448-1603/OL799306 b | [(Varela et al., 2021)](https://sciwheel.com/work/citation?ids=9115112&pre=&suf=&sa=0&dbf=0) |
| PRRSF  PRRSR  Probe PRRS | GTAGTYGCRCTCCTTTGGGGRGTGT  GACGCCGRACGASAAAYGCGTGGTTA  FAM-TACATTCTGGCCCCTGCCCAYC-TAMRA | 173 | 14607-/MN642104c | [(Kleiboeker et al., 2005)](https://sciwheel.com/work/citation?ids=6876380&pre=&suf=&sa=0&dbf=0) |

^a-c^ Gen Bank reference sequence of each virus.

**Table S2.** Information about PPV2 sequences obtained from PPV2-positive lung tissue samples from three regions with high swine production in Colombia.

| **Sequence**  **Number** | **Region of origin** | **Collection date** | **GenBank**  **accession number** | **Clinical syndrome** |
| --- | --- | --- | --- | --- |
| 1 | Valle | March 4, 2021 | ON210855 | Cough, dyspnea |
| 2 | Antioquia | February 3,2021 | ON210856 | Cough |
| 3 | Antioquia | October 20,2020 | ON210857 | Cough, dyspnea |
| 4 | Antioquia | October 20,2020 | ON210858 | Stillbirth |
| 5 | Cundinamarca | November20, 2020 | ON210859 | Cough, sneezing |
| 6 | Cundinamarca | November 20, 2020 | ON210860 | Cough, sneezing |
| 7 | Cundinamarca | March 23, 2021 | ON210861 | Cough, dyspnea, wasting |
